# Following the Science to Generate Well-Being: Using the Highest-Quality Experimental Evidence to Design Interventions

**DOI:** 10.3389/fpsyg.2021.739352

**Published:** 2021-12-15

**Authors:** Stewart I. Donaldson, Victoria Cabrera, Jaclyn Gaffaney

**Affiliations:** Department of Psychology, Claremont Graduate University, Claremont, CA, United States

**Keywords:** positive psychology intervention, well-being, randomized controlled trial, systematic review, exemplar method

## Abstract

The second wave of devastating consequences of the COVID-19 pandemic has been linked to dramatic declines in well-being. While much of the well-being literature is based on descriptive and correlational studies, this paper evaluates a growing body of causal evidence from high-quality randomized controlled trials (RCTs) that test the efficacy of positive psychology interventions (PPIs). This systematic review analyzed the findings from 25 meta-analyses, 42 review papers, and the high-quality RCTs of PPIs designed to generate well-being that were included within those studies. Findings reveal PPIs have the potential to generate well-being even during a global pandemic, with larger effect sizes in non-Western countries. Four exemplar PPIs—that have been tested with a high-quality RCT, have positive effects on well-being, and could be implemented during a global pandemic—are presented and discussed. Future efforts to generate well-being can build on this causal evidence and emulate the most efficacious PPIs to be as effective as possible at generating well-being. However, the four exemplars were only tested in WEIRD (Western, Educated, Industrial, Rich, and Democratic) countries but seem promising for implementation and evaluation in non-WEIRD contexts. This review highlights the overall need for more rigorous research on PPIs with more diverse populations and in non-WEIRD contexts to ensure equitable access to effective interventions that generate well-being for all.

## Introduction

In response to psychology’s strong emphasis on pathology and repairing human deficits, Seligman and Csikszentmihalyi (2000) provided a vision for the next generation of psychological scientists to spend at least some of their careers understanding the factors that make life worth living and preventing pathologies that arise when life is barren and meaningless. The call was answered and thousands of peer-reviewed articles on positive psychology topics have been published and more than a thousand of these articles included empirical tests of positive psychology theories, principles, and interventions (Donaldson et al., 2015; Kim et al., 2018). Furthermore, this new science is now being conducted across many disciplines and professions, five continents, and more than 60 countries (Kim et al., 2018; Donaldson et al., 2020). It is hard to believe anyone could have imagined that two decades of rigorous peer-reviewed positive psychological science would become one of the key knowledge bases that could be used to generate well-being in the unprecedented global pandemic of 2020–2021.

“Follow the Science” is the cry being heard from public health scientists around the world as they try to stop the spread of COVID-19 by “flattening the curve.” It is the same cry that we hear with respect to finding treatments to reduce the severity and length of illness caused by the coronavirus, as well as developing effective vaccines. Scientists working on each of these public health challenges are using the best science they have available to prevent the spread of the virus, find effective treatments, and develop vaccines that can be taken to scale to alleviate the fear, trauma, and suffering occurring across the globe (National Institutes of Health, 2020; World Health Organization, 2020).

The second wave of devastating consequences of this global pandemic has been linked to dramatic declines in well-being (see Panchal et al., 2020). These undesirable consequences have affected marginalized and vulnerable groups disproportionately, increasing health and economic disparities around the world (United Nations, 2020). In the same way, public health scientists are following the most rigorous science available to combat the physical health impacts of the virus; it is also important to follow the most rigorous positive psychology intervention (PPI) science to design new PPIs that can be implemented during the pandemic to generate well-being across the globe (see Seligman, 2008; Donaldson et al., 2020). Just as equitable access to effective vaccines has been a major concern worldwide, it is also critical to ensure access and efficacy of PPIs beyond WEIRD (Western, Educated, Industrial, Rich, and Democratic) contexts to ensure that they generate well-being for all.

### Present Study

The focus of this review is to identify the most promising PPIs for generating well-being by identifying and describing the most efficacious PPIs to date as determined by high-quality randomized controlled trials (RCTs). Like the health scientists designing RCTs to test the efficacy of treatments and vaccines to combat the coronavirus, we are using a conceptual version of the exemplar method (Bronk, 2012; Bronk et al., 2013) to intentionally identify the most efficacious PPIs when tested by the rigorous RCTs. The following sections will describe how we evaluated findings from reviews, meta-analyses, and peer-reviewed RCTs testing PPI efficacy in order to determine the most promising exemplar PPIs for generating well-being during the global pandemic. We hope the findings will help intervention researchers and practitioners around the world, including those in non-WEIRD contexts, to optimize the design and implementation of future PPIs.

## Materials and Methods

**Figure 1:**
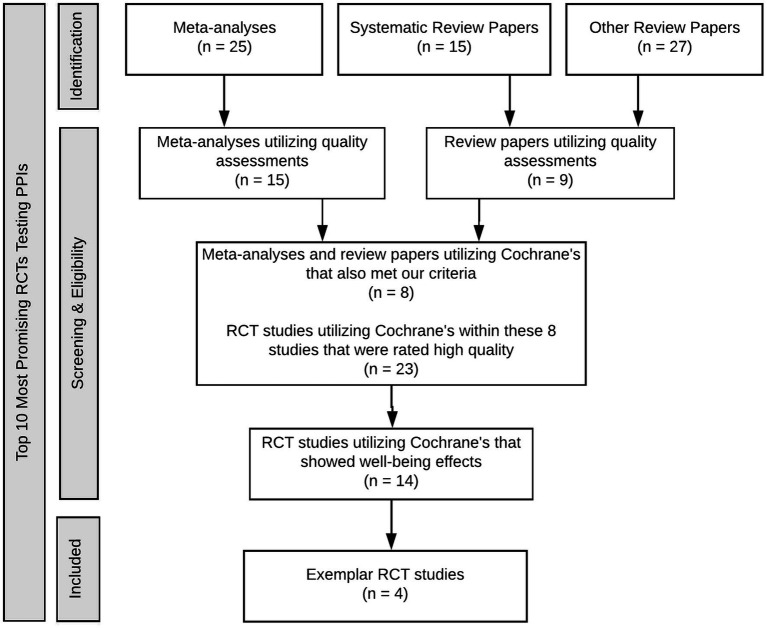
Flow diagram of the search and selection procedure for target studies.

The exemplar method is a research approach that involves focusing a study on a select sample that exemplifies the area of interest (Bronk, 2012; Bronk et al., 2013). In the spirit of positive psychology, researchers can study within the upper bounds of what is possible as opposed to limiting study to the averages of what is typical. For this reason, the method has been utilized in many previous positive psychology studies (e.g., Reimer et al., 2009; Dunlop et al., 2012; Reimer and Reimer, 2015; Morton et al., 2019). In this study, high-quality RCTs were chosen as exemplars from a larger pool of studies previously published in meta-analyses and review papers. The subsequent sections outline the search strategy, selection, and coding processes. Refer to Figure 1 for a flow diagram of the exemplar process utilized for this study.

### Search Strategy

A systematic literature search was conducted for meta-analyses and review papers in the following five databases: Academic Search Premier, PsycINFO, PsycArticles, PubMed, and Scopus, covering the period from 1998 (the start of the positive psychology movement) to 2020. The last run was conducted on June 3, 2021. In addition, a hand search was conducted through the websites of three non-Western journals in the field of positive psychology: the Indian Journal of Positive Psychology, the Iranian Journal of Positive Psychology, and the Middle East Journal of Positive Psychology.

### Selection of High-Quality RCTs Testing PPIs

The present study focused its in-depth analysis on PPIs that were examined by RCTs that had undergone quality assessment by previous peer-reviewed meta-analyses and reviews. The 25 meta-analyses and 42 review articles were reviewed for the use of quality assessments (QA). Fifteen meta-analyses and nine review papers utilized some sort of QA, the most common of which was Cochrane’s Tool for Assessing Risk of Bias (Higgins et al., 2011). From eleven papers that used Cochrane’s, after removing two for calculating ratings differently from the others (Brown et al., 2019; Carrillo et al., 2019) and one that did not have studies meeting inclusion criteria (Macaskill and Pattison, 2016), we analyzed the QA ratings within the eight remaining papers (Bolier et al., 2013; Sutipan et al., 2016; Weiss et al., 2016; Chakhssi et al., 2018; Hendriks et al., 2018; Carr et al., 2020; Hendriks et al., 2020; Tejada-Gallardo et al., 2020).

The studies were included based on the following criteria: (1) included in one of the aforementioned meta-analyses or review papers, (2) utilized some form of a Cochrane’s quality assessment rating, (3) RCTs using individual random assignment, (4) intervention described as a “PPI,” “positive psychological intervention,” a “positive intervention”, “positive psychotherapy”, “well-being intervention” or referred to their work in the context of “positive psychology” (in order to minimize bias by relying on an explicit, objective criterion for inclusion), (5) published in a peer-reviewed journal in the English language, and (6) intervention focused on improvement of psychological or mental well-being or any dimension based on any one of five major definitions: Subjective Well-being (SWB; Diener, 1984), the PERMA model of flourishing (PERMA; Seligman, 2011), Thriving (Su et al., 2014), Psychological Well-being (PWB; Ryff, 1989), and Quality of Life (Seligman and Csikszentmihalyi, 2000). As part of our exemplar approach, a variety of PPI types and features, samples, and well-being measures were included to ensure the sample was representative of the wide variety of PPI RCTs in the literature. We excluded: (1) cluster RCTs and quasi-experimental studies. (2) interventions that were not described as a PPI, positive psychological intervention, positive intervention, positive psychotherapy, well-being intervention, or referred to their work in the context of positive psychology (3) studies that solely measured as an outcome the reduction of depression, anxiety, or other negative emotions or states without a psychological well-being component as an outcome. (4) PPIs that solely changed behavior without a psychological well-being component as an outcome (e.g., physical activity, and cessation of smoking, etc.). (5) studies published in book chapters, dissertations, and grey literature, and (6) articles not published in the English language.

After removing duplicates and studies that did not meet our inclusion criteria, 23 high-quality studies were identified within the eight papers that met Cochrane’s high versus low or moderate-quality criteria (source of bias criteria: random sequence generation, allocation concealment, blinding of participants and personnel, blinding of outcome assessment, incomplete outcome data, selective reporting, and anything else; Higgins et al., 2011). Of these 23 studies that were rated as high-quality, six were removed because they received different quality ratings from different papers. Out of the 17 remaining studies, 14 of the most promising trials of PPIs were identified where the PPI was found to improve at least one well-being outcome.

### Coding of High-Quality RCT PPIs

Coding was conducted for: (1) year, (2) country of origin, (3) setting of intervention, (4) participants, (5) mention of PP, (6) PPI term used, (7) PPI description, (8) theory behind the intervention, (9) control group type, (10) time points assessed, (11) well-being measure, (12) other non-well-being measures, (13) well-being outcomes & effect sizes, (14) other non-well-being outcomes & effect sizes, (15), other findings, (16), the review or meta-analysis that assessed its quality, (17) applicable in the context of a global pandemic, (18) relevant for equity or marginalized groups heavily impacted by COVID-19, (19) relevant for vulnerable populations during a global pandemic, and (20) and each of the previously assessed quality assessments and quality ratings that used Cochrane’s.

### Selection of Four Exemplar PPIs

Fourteen promising PPIs were further analyzed to find exemplar PPIs according to the following criteria: (1) improved well-being outcomes with medium to large effect sizes; (2) outcomes were attributed with confidence to the PPI; and (3) relevance in a global pandemic where the PPI could be delivered at scale and at a lower cost (e.g., online), could be delivered in social distancing conditions (e.g., delivered remotely *via* online or phone), and incorporated flexible delivery or content (e.g., participants choose their own content or time to complete it).

## Results

### Systematic Reviews and Meta-Analyses

The science of PPIs has matured to the point where we now have numerous systematic reviews and meta-analyses (see Table 1). There has also been a surge in recent studies testing PPIs (e.g., Biswas-Diener, 2020).

**Table 1:** Positive psychology intervention meta-analyses.

References	Title	Sample	Main Effect Findings	Moderator Findings
Van Agteren et al. (2021)	A systematic review and meta-analysis of psychological interventions to improve mental wellbeing	393 studies, 53,288 participants from clinical, non-clinical and physical illness populations in 42 countries	Multi-component PPIs were effective with small to moderate effects on overall well-being for the general population (Hedge’s *g* = 0.28), mentally ill population (*g* = 0.37), and physically ill population (*g* = 0.52). See the article for effects for ACT, Compassion, CBT, expressive writing, mindfulness, multi-theoretical, singular PPI, and reminiscence interventions.	Moderators that increased effectiveness in well-being included: time to follow-up (shorter versus longer, with effect sizes maintained at the 3-month follow-up but dropping at 6 months), as well as comparison groups (waitlist-control or assessment-only design versus placebo).
Carr et al. (2020)	Effectiveness of positive psychology interventions: a systematic review and meta-analysis	347 studies, over 72,000 participants from clinical and non-clinical child and adult populations in 41 countries	PPIs with an average of ten sessions over six weeks offered in multiple formats and contexts were effective with small to medium effects on well-being (Hedge’s g = 0.39), strengths (*g* = 0.46), quality of life (g = 0.48), depression (*g*−0.39), anxiety (*g* = −0.62), and stress (*g* = −0.58), with gains maintained at three months follow-up.	Moderators that increased effectiveness of well-being included: life-stage (older versus younger), clinical status (clinical problems versus not), recruitment method (referred versus self-selected), country (individuals in non-western countries versus western), program format (engagement in longer individual or group therapy programs versus self-help), program type (containing two or more PPIs versus one PPI), program duration (longer versus shorter), control group type (no intervention type comparison group versus alternate intervention type), PPI type (e.g., savoring, optimism, and hope versus forgiveness and goal-setting), and alternative intervention type (PPIs versus treatment-as-usual or CBT), study quality (lower versus higher quality), and year of publication (older versus newer).
Geerling et al. (2020)	The effect of positive psychology interventions on well-being and psychopathology in patients with severe mental illness: A systematic review and meta-analysis	16 studies (including 9 RCTs), 729 patients	PPIs in people with severe mental illness were not effective on well-being or psychopathology in comparison to control conditions. However, when only looking at within-group effects, these PPIs were effective with moderate effects on well-being (*g* = 0.40) and with a large effect on psychopathology (*g* = 0.70).	Moderators for well-being included diagnosis (patients with major depressive disorder over schizophrenia or mixed samples). Moderators showed no significant differences between sub-groups for treatment duration or format.
Heekerens and Eid (2020)	Inducing positive affect and positive future expectations using the best-possible-self intervention: A systematic review and meta-analysis	34 RCT studies, 4,462 participants	The Best Possible Self interventions were effective PPIs with small effects for positive affect (*g* = 0.28) and optimism (*g* = 0.21), with no substantial follow-up effects.	Moderators included: assessment of momentary affect immediately after the intervention and conceptualizing optimism as positive future expectations instead of a general orientation in life.
Hendriks et al. (2020)	The efficacy of multi-component positive psychology interventions: A systematic review and meta-analysis of randomized controlled trials	50 RCT studies in 51 articles, 6,141 participants	Multi-component PPIs (MPPIs) were effective with small effects for subjective well-being (*g* = 0.34) and depression (*g* = 0.29), small to moderate effects for psychological well-being (*g* = 0.39) and anxiety (*g* = 0.35), and moderate effects for stress (*g* = 0.48), after taking study quality and outliers into account.	Moderators included region and study quality. Non-Western countries and lower-quality studies found greater effects.
Koydemir et al. (2020)	A meta-analysis of the effectiveness of randomized controlled positive psychological interventions on subjective and psychological well-being	68 RCT studies of nonclinical populations, 16,085 participants	PPIs were effective with small effects for psychological well-being (Cohen’s *d* = 0.08) and subjective well-being (*d* = 0.22), with small to moderate effects when targeting both types of well-being (*d* = 0.43), with evidence for sustained effects at follow-up.	Moderators included: longer interventions (versus shorter), traditional methods (versus technology-assisted methods), and mixed outcomes for age.
Tejada-Gallardo et al. (2020)	Effects of school-based multicomponent positive psychology interventions on well-being and distress in adolescents: A systematic review and meta-analysis	9 studies in 9 articles, 4,898 participants	Multi-component PPIs (MPPIs) were effective with small effects for subjective well-being (*g* = 0.24), psychological well-being (*g =* 0.25), and depression symptoms (*g* = 0.28).	Moderators included: year of publication (more recent over older), study design (non-randomized over randomized), type of intervention (multi-component combined with another type of positive intervention), control group (placebo over waitlist), quality of studies (removing low-quality studies lowered effects for subjective well-being and raised effect size for psychological well-being and depression symptoms), and measurement of follow-up (no-followup over follow-up).
Brown et al. (2019)	The effects of positive psychological interventions on medical patients’ anxiety: A meta-analysis	12 RCT studies with 1,131 participants; 11 non-randomized trials with 300 participants	PPIs were effective with small to medium effects for patient anxiety (*g* = −0.34), sustained 8 weeks post (*g* = −0.31).	Moderators included: clinician-led interventions (versus self-administered), longer interventions (versus shorter).
Carrillo et al. (2019)	Effects of the Best Possible Self intervention: A systematic review and meta-analysis	29 studies in 26 articles, 2,909 participants	The Best Possible Self (BPS) interventions were effective PPIs with small effects for negative affect (d+ = 0.192), and depressive symptoms (d+ = 0.115), as well as moderate effects for positive affect (d+ = 0.511), optimism (d+ = 0.334), and well-being (d+ = 0.325).	Moderators included: older participants and shorter (total minutes of) practice. BPS was more effective than gratitude interventions for positive and negative affect outcomes.
Donaldson et al. (2019a)	Evaluating positive psychology interventions at work: A systematic review and meta-analysis	22 studies, 52 independent samples, 6,027 participants from 10 countries	Five workplace PPIs (psychological capital, job crafting, strengths, gratitude, and employee well-being) can be effective with small effects for desirable work outcomes (performance, job well-being, engagement, etc.; *g* = 0.25) and with small to moderate effects for undesirable work outcomes (negative performance, negative job well-being; *g* = −0.34).	Moderators for both desirable and undesirable outcomes did not include: type of theory or intervention delivery method.
Howell and Passmore (2019)	Acceptance and Commitment Training (ACT) as a positive psychological intervention: A systematic review and initial meta-analysis regarding ACT’s role in well-being promotion among university students	5 randomized experiments of university students, 585 participants	Acceptance and Commitment Training (ACT) was an effective PPI with small effects on well-being (*d* = 0.29).	N/A
Lomas et al. (2019)	Mindfulness-based interventions in the workplace: An inclusive systematic review and meta-analysis of their impact upon wellbeing	35 RCT studies, 3,090 participants	Mindfulness-based interventions (MBIs) were effective with moderate effects for stress (Standardized Mean Difference = −0.57), anxiety (SMD = −0.57), distress (SMD = −0.56), depression (SMD = −0.48), and burnout (SMD = −0.36), as well as small to moderate effects for health (SMD = 0.63), job performance (SMD = 0.43), compassion (SMD = 0.42), empathy (SMD = 0.42), mindfulness (SMD = 0.39), and positive well-being (SMD = 0.36), with no effects for emotional regulation.	Moderators for health included: region (higher effects for studies in North America), intervention type (MBSR versus other intervention types), and age (younger versus older). Moderators for positive well-being and compassion included: gender (more women in the intervention group).
Slemp et al. (2019)	Contemplative interventions and employee distress: A meta-analysis	119 studies, 6,044 participants	Contemplative interventions (e.g., mindfulness, meditation, and other practices) were effective in RCTs with small to moderate effects for reducing employee general distress (*d* = 0.39), sustained at follow-up. More specifically, distress consisted of anxiety (*d* = 0.58), negative affect (*d* = 0.50), stress (*d* = 0.47) depression (*d* = 0.42), somatic symptoms (*d* = 0.40), and burnout (*d* = 0.20).	Moderators included: type of contemplative intervention (highest for general meditation-based interventions, followed by mindfulness-based and ACT-based interventions) and type of control group (no-intervention or comparisons that received no education only versus active control comparisons). Moderators did not include: study quality ratings, overall duration of the programs, or the number of sessions included. Adjustments for publication bias lowered overall effects.
White et al. (2019)	Meta-analyses of positive psychology interventions: The effects are much smaller than previously reported	2 previous meta-analyses (Sin and Lyubomirsky, 2009; Bolier et al., 2013)	When small sample size bias was taken into account, PPIs were effective with small effects for well-being (*r* = .10), with variable mixed effectiveness for depression.	Study notes need for increasing sample sizes in future studies.
Chakhssi et al. (2018)	The effect of positive psychology interventions on well-being in clinical populations: A systematic review and meta-analysis	30 studies, 1,864 participants with clinical disorders	PPIs were effective with small effects for well-being (*g* = 0.24) and depression *(g* = 0.23), moderate effects for anxiety (*g* = 0.36), and no significant effects for stress, with similar effects 8 to 12 weeks post.	Moderator for well-being included: guided PPIs (versus unguided, such as self-help). Moderator for stress included: control group type (no intervention/waitlist control versus active or treatment-as-usual control). Moderators did not include: population type (psychiatric versus somatic disorders), intervention format (individual versus group), intervention duration (shorter versus longer), or type of PPI (PPI therapy programs versus single PPIs).
Curry et al. (2018)	Happy to help? A systematic review and meta-analysis of the effects of performing acts of kindness on the well-being of the actor	27 studies in 24 articles, 4,045 participants	Kindness interventions (e.g., random acts of kindness) were effective PPIs with small to medium effects for well-being (for the actor of kindness; δ = 0.28).	Moderators did not include: sex, age, type of participant, intervention, control condition, or outcome measure.
Hendriks et al. (2018)	The efficacy of positive psychology interventions from non-Western countries: A systematic review and meta-analysis	28 RCT studies, 3,009 participants	PPIs from non-Western countries were effective with moderate effects for subjective wellbeing (*g* = 0.48) and psychological wellbeing (*g* = 0.40), and a large effect on depression (*g* = 0.62) and anxiety (*g* = 0.95).	Moderators did not include: study population (clinical or non-clinical), mode of delivery of the PPI (group or self-help), intervention type (single component or multi-component), type of control group (active/placebo or non-active/waitlist), duration of the intervention (≤ 8 weeks or > 8 weeks), or cultural adaptation of the PPI (yes or no).
Dhillon et al. (2017)	Mindfulness-based interventions during pregnancy: A systematic review and meta-analysis	14 articles (some RCT and some non-RCT studies), pregnant (prenatal) participants	Mindfulness-based interventions showed no significant effects for anxiety, depression, or perceived stress in the pooled RCTs, but each showed a significant effect in the pooled non-RCTs: anxiety (SMD = −0.48), depression (SMD = −0.59), and perceived stress (SMD = −3.28). Further, mindfulness as an outcome showed significant effects for both the pooled RCT (SMD = −0.57) and pooled non-RCT studies (SMD = −0.60).	N/A
Dickens (2017)	Using gratitude to promote positive change: A series of meta-analyses investigating the effectiveness of gratitude interventions	38 studies, 5,223 participants	Gratitude interventions can be effective with small to medium effects for well-being, happiness, life satisfaction, grateful mood, grateful disposition, positive affect, and depressive symptoms, with mixed findings for negative affect and stress, and no significant effects for physical health, sleep, exercise, prosocial behavior, or self-esteem. Please see the full paper for effect sizes for each of the comparison group types: neutral, positive, and negative conditions.	Moderators included: adults (versus children or college-aged). Moderators did not include: gender, type of neutral comparison group, duration of the follow-up period.
Davis et al. (2016)	Thankful for the little things: A meta-analysis of gratitude interventions	32 studies in 26 articles	Gratitude interventions were effective PPIs with small effects for psychological well-being (*d* = 0.31) but not gratitude as an outcome itself (*d* = 0.20) in comparison to measurement-only controls. However, gratitude interventions were effective with moderate effects for gratitude (*d* = 0.46) and small effects for psychological well-being (*d =* 0.17), with no significant effects for anxiety (*d* = 0.11), in comparison to alternate-activity conditions.	Moderators did not include: type of gratitude intervention or dosage (neither days nor minutes of participation).
Weiss et al. (2016)	Can we increase psychological well-being? The effects of interventions on psychological well-being: A meta-analysis of randomized controlled trials	27 RCT studies, 3,579 participants	Behavioral interventions were effective with moderate effects for psychological well-being (*d* = 0.44), with small effects at follow-up (*d =* 0.22).	Moderators included: clinical groups (versus non-clinical) and individual face-to-face interventions (versus self-help or group face-to-face). Moderators did not include: age, number of sessions, measurement instrument, and control group. Lower-quality studies found greater effects.
Theeboom et al. (2014)	Does coaching work? A meta-analysis on the effects of coaching on individual-level outcomes in an organizational context	18 studies, 2,090 participants, organizational context	Coaching was effective with moderate to large effects for goal-directed self-regulation (*g* = 0.74) and with small to moderate effects for performance/skills (*g* = 0.60), well-being (*g* = 0.46), coping (*g* = 0.43), and work attitudes (*g* = 0.54), in an organizational context.	N/A
Bolier et al. (2013)	Positive psychology interventions: A meta-analysis of randomized controlled studies	39 RCT studies in 40 articles, 6,139 participants	PPIs were effective with small effects for subjective well-being (SMD = 0.34), psychological well-being (SMD *=* 0.20), and depression (SMD *=* 0.23).	Moderators for decreasing depression included: longer duration (four or eight weeks versus as opposed to less than four weeks), recruited as a referral from a healthcare practitioner or hospital (versus recruitment at a community center, online, or at a university), the presence of psychosocial problems, and individual delivery (versus self-help or group). Lower-quality studies found greater effects.
Mazzucchelli et al. (2010)	Behavioral activation interventions for well-being: A meta-analysis	20 RCT studies, 1,353 participants	Behavioral Activation (BA) interventions were effective with moderate effects for well-being (*g* = 0.52) in both non-clinical participants and those with depressive symptoms, indicating that BA can be useful for non-clinical populations alongside its more common setting as a treatment for depression.	N/A
Sin and Lyubomirsky (2009)	Enhancing well-being and alleviating depressive symptoms with positive psychology interventions: A practice-friendly meta-analysis	51 studies, 4,266 participants	PPIs were effective with moderate effects for well-being (mean *r* = .29) and depressive symptoms (mean *r* = .31).	Moderators included: self selection to participate in the PPI, older age (versus younger), depression status, individual therapy (versus group), and relatively longer duration (versus shorter).

*Small to moderate effects were characterized by the following benchmarks for Hedge’s g, Cohen’s d, SMD, and δ: small = 0.2, medium = 0.5, large = 0.8, and for r: small = .1, moderate = .3, and large = .5 (**Cohen, 1988**)*.

#### Well-Being Effect Sizes

Most of these reviews and meta-analyses of RCTs show that PPIs, on average, do have at least small to medium-sized positive effects on important outcomes, such as well-being (Table 2). By way of example, Van Agteren et al. (2021) and Hendriks et al. (2020) both examined the efficacy of multi-component positive psychology interventions (MPPIs). Van Agteren et al. (2021) found small to moderate effects on overall well-being for the general, mentally ill, and physically ill populations. Hendriks et al. (2020) concluded that MPPIs studies had an overall small effect on subjective well-being and depression, and a small to moderate effect on psychological well-being. In addition, they suggest MPPIs had an overall small to moderate effect on anxiety and a moderate effect on stress. Further, Donaldson et al. (2019a) published a meta-analysis of the most rigorous PPI studies conducted in the workplace. They found that the workplace interventions had small to moderate positive effects across both desirable and undesirable work outcomes (e.g., job stress), including well-being, engagement, leader-member exchange, organization-based self-esteem, workplace trust, forgiveness, prosocial behavior, leadership, and calling.

**Table 2:** Small to moderate well-being effect sizes in positive psychology intervention meta-analyses.

Outcome	Effect size
Well-being	*g* = 0.28 (Van Agteren et al., 2021) *g* = 0.39 (Carr et al., 2020) *g* = 0.40 (Geerling et al., 2020) d+ = 0.325 (Carrillo et al., 2019) SMD = 0.36 (Lomas et al., 2019) *r* = .10 (White et al., 2019)*δ* = 0.28 (Curry et al., 2018) *g* = 0.24 (Chakhssi et al., 2018; Dickens, 2017) *g* = 0.46 (Theeboom et al., 2014) *g* = 0.52 (Mazzucchelli et al., 2010) mean *r* = .29 (Sin and Lyubomirsky, 2009)
Subjective well-being	*g* = 0.34 (Hendriks et al., 2020) d = 0.22 (Koydemir et al., 2020) *g* = 0.24 (Tejada-Gallardo et al., 2020) *g* = 0.48 (Hendriks et al., 2018) SMD = 0.34 (Bolier et al., 2013)
Psychological well-being	*g* = 0.39 (Hendriks et al., 2020) d = 0.08 (Koydemir et al., 2020) *g* = 0.25 (Tejada-Gallardo et al., 2020) *g* = 0.40 (Hendriks et al., 2018) *d* = 0.31 (Davis et al., 2016) *d* = 0.44 (Weiss et al., 2016) SMD = 0.20 (Bolier et al., 2013)

#### Moderators

These meta-analyses based on numerous empirical tests and thousands of participants illustrate the conditions under which PPIs can generate well-being and positive human functioning. Many moderators were tested and found to impact effect size. Each of these meta-analyses focused on different types of PPIs with differing study designs, which explains some variability in findings.

Some features of the PPIs were found to be moderators that impacted effect sizes, including the program format, program type, and duration, but not frequency. The program format showed that individualized interventions led to greater effects than self-help or group (Sin and Lyubomirsky, 2009; Weiss et al., 2016; Carr et al., 2020). The program type findings were mixed with Carr et al. (2020) demonstrating that multi-component PPIs showed greater effects than single component PPIs, but Hendriks et al. (2018) showed no effect for this moderator. The impacts of duration were also mixed with Carr et al. (2020), Koydemir et al. (2020), and Sin and Lyubomirsky (2009) finding that longer interventions led to greater effects, but Carrillo et al. (2019) found the opposite and Davis et al. (2016), Geerling et al. (2020), Hendriks et al. (2018), and Slemp et al. (2019) not finding this effect. Frequency was only tested by Slemp et al. (2019) study of contemplative interventions and was not found to be a moderator.

Features of the participants were also found to be moderators, including age, gender, and clinical status. Many meta-analyses found age to be a moderator, with older participants showing a larger effect size than younger participants (Sin and Lyubomirsky, 2009; Dickens, 2017; Curry et al., 2018; Carrillo et al., 2019; Carr et al., 2020). However, Weiss et al. (2016) did not report this finding. Gender was a moderator in one meta-analysis with women showing greater effects (Lomas et al., 2019) but was not found to be a moderator by Curry et al. (2018) or Dickens (2017). Meta-analyses that compared clinical participants to non-clinical participants found that clinical participants demonstrated greater effects (Sin and Lyubomirsky, 2009; Weiss et al., 2016; Carr et al., 2020). However, Hendriks et al. (2018) did not replicate this finding.

Features of the study were also moderators, including country of study, study quality, and control group type, in that non-Western countries, lower-quality studies, and those that used no intervention as a comparison group tended to report higher effect sizes. Hendriks et al. (2018) found that PPIs from non-Western countries tend to report larger effect sizes than those from Western countries with the caveat that these studies tend to have lower study quality. Supporting these findings, Carr et al. (2020) and Hendriks et al. (2020) both found country as a moderator of well-being, with individuals in non-WEIRD countries showing greater effects than those in WEIRD countries (Henrich et al., 2010). However, both meta-analyses rated few non-WEIRD studies as good or high-quality according to their quality assessments: 5 out of 64 non-WEIRD studies in Carr et al. (2020) and 1 out of 13 non-WEIRD studies in Hendriks et al. (2020). Across the two meta-analyses, only 19.35% of studies took place in non-WEIRD countries; 48.05% of non-WEIRD studies were rated poor quality, 44.16% of non-WEIRD studies were rated fair/moderate quality, and only 7.79% of non-WEIRD studies were rated good/high quality. Beyond country distinctions, lower-quality RCTs often overestimated the effects of PPIs (e.g., Carr et al., 2020; Hendriks et al., 2020; Tejada-Gallardo et al., 2020). However, this finding was not found by Slemp et al. (2019). As further indication of study quality, the type of control group was also found to be a moderator where studies that used no intervention as a comparison group led to greater effects than those that used an alternative/active intervention; plus, those that used a placebo led to greater effects than those that used a waitlist design (Sin and Lyubomirsky, 2009; Dickens, 2017; Curry et al., 2018; Carrillo et al., 2019; Slemp et al., 2019; Carr et al., 2020; Tejada-Gallardo et al., 2020). However, these control group findings were not replicated by all meta-analyses (Weiss et al., 2016; Dickens, 2017; Curry et al., 2018; Hendriks et al., 2018). Lastly, the study recruitment method showed mixed results with Carr et al. (2020) finding that those with participants who were referred to the study had greater effects than those with participants who self-selected into it, yet Sin and Lyubomirsky (2009) found the opposite.

#### Small Sample Bias

One criticism of some of these meta-analyses is that they are limited by small sample bias. For example, White et al. (2019) reanalyzed two highly cited meta-analyses (Sin and Lyubomirsky, 2009; Bolier et al., 2013) and corrected their findings for small sample size bias. While the effect sizes of PPIs on well-being were smaller (approximately *r* = 0.10) after the adjustment, both meta-analyses still demonstrated a statistically significant improvement of well-being (White et al., 2019).

### Exemplar PPIs

The fourteen promising PPIs identified by this review based on the highest-quality RCTs were all conducted in WEIRD countries. Overall, there were fewer studies from non-WEIRD countries in the sample analyzed for this review and they were of moderate or low quality so were not included in the most promising PPIs. Out of the highest-quality interventions, four exemplars were identified as the most promising PPIs for generating well-being in a global pandemic (see Table 3). All of the Four Most Promising PPIs were conducted with adult samples. Three of the promising PPIs used samples relevant to vulnerable populations during a pandemic: individuals with low to moderate well-being (Schotanus-Dijkstra et al., 2017) mild to moderate depression (Ivtzan et al., 2016), and stressed employees (Feicht et al., 2013).

**Table 3:** Promising PPIs for generating well-being in a global pandemic.

Reference, Country	Drozd et al. (2014), Norway	Feicht et al. (2013), Germany	Ivtzan et al. (2016), UK	Schotanus-Dijkstra et al. (2017), Netherlands
Sample	Healthy adults	Employees experiencing stress and high work demands at an insurance company	Healthy and mildly depressed adults (educators, office workers, meditators)	Adults with low/moderate well-being
PPI	“Better Days” multi-component training	Online multi-component “happiness training” for employees	Positive Mindfulness Program – mindfulness and positive psychology training	Multi-component, guided “positive self-help intervention” with email support
Delivery	Online	Online	Online	In person, online
Sessions, Duration	13 10-min sessions, 4 weeks	10–15 min weekly for 7 weeks	Approximately 30 minutes for each of 8 weekly sessions weekly over 8 weeks	Four hours per week for each of 9 weekly sessions, over 9 to 12 weeks
Assessment	Pre, post at 1-month, 2-month and 6-month follow-ups after intervention onset	Pre, post at 7 weeks, 4-week follow-up	Pre, post at 8 weeks, 1-month follow-up	Pre, post at 3 months, 6-month and 12-month follow-up
Topics Covered	Gratitude, engagement and pleasant activities, character strengths, acts of kindness, gratitude, mastery and reattribution, optimism, flow, gratitude, adaptation and attribution, stress and mindfulness	Gratitude, positive relationships, mindfulness, flow, strengths, good deeds, joy	Self-awareness, positive emotions, self-compassion, self-efficacy, strengths, autonomy, meaning, positive relationships, engagement (savoring)	Positive emotions, discovering strengths, use of strengths, flow, optimism, hope, self-compassion, resilience, positive relations
Well-being Measures	SHS, PANAS, LOT-R	VAS, WHO-5, FS	PHI, GQ6, SCS-short, APWB, GSE, MLQ-P, COS	MHC-SF, FS
Well-Being Outcomes	Improved happiness at post: *d* = 0.65 (medium) Improved ratio of positive to negative affect at post, and 2- and 6-month follow-ups. No significance for optimism as a mediator.	Improved Happiness: Post: *d* = 0.93 (large) 4wks: *d* = 0.92 (large) Improved Satisfaction: Post: *d* = 1.17 (large) 4wks: *d* = 1.10 (large) Improved Flourishing: Post: *d* = 0.42 (medium) 4wks: *d* = 0.25 (small) Improved Quality of Life: Post: *d* = 1.06 (large) 4wks: *d* = 0.94 (large)	Improved at one-month follow-up): Well-being: ηp2 = 0.124 (medium) Gratitude: ηp2 = 0.083 (medium) Self-compassion: ηp2 = 0.165 (medium) Compassion for others at post only. No significance for self-efficacy.	Improved well-being: Post: *d* = 0.68 (medium) 6-month: *d* = 0.66 (medium) No significance for flourishing.
Other Measures	N/A	REQ, SWS, ANT	BDI-II, PSS	HADS- D; HADS-A
Other Outcomes	N/A	Reduced perceived stress at post, no significance in recovery experience, saliva, or attention networks.	Decreased perceived stress and depression at post and 1-month follow-up.	Decreased anxiety and depression at 3-month post, and 6-month and 12-month follow-ups.
Control Group	Waitlist	Waitlist	Waitlist	Waitlist

*Well-being measures: APWB, Psychological Well-being Autonomy Subscale; COS, Compassion For Others Scale; FS, The Flourishing Scale; GQ6: Gratitude Questionnaire, 6-item Form; GSE, Generalised Self-efficacy Scale; LOT-R, Life Orientation Test-Revised; MHC-SF, Mental Health Continuum-Short Form; MLQ-P, Meaning in Life Questionnaire-Presence Subscale; PANAS, Positive and Negative Affect Schedule; PHI, Pemberton Happiness Index; SCS-short, Self-compassion Scale; SHS, Subjective Happiness Scale; VAS, Visual Analog Scale; and WHO-5, WHO Well-being Index*.

*Other measures: ANT, Attention Network Test; BDI-II, Beck’s Depression Inventory-II; HADS-A, Hospital Anxiety and Depression Scale-Anxiety Subscale; HADS- D, Hospital Anxiety and Depression Scale-Depression Subscale; PSS, Perceived Stress Scale; REQ, Recovery Experience Questionnaire; SPT, Subjective Probability Task; and SWS, The Stress Warning Signals Scale*.

The four exemplar PPIs are all MPPIs that focus on training, improved well-being with medium to large effect sizes, and can be feasibly implemented during a global pandemic and beyond. The most popular topics were strengths, gratitude, positive relationships, positive emotions, and mindfulness. A variety of measures were used to measure well-being. This variety reflects the lack of consensus on a universal definition of well-being in the positive psychology literature (Diener et al., 2018), which can make it challenging to compare the impact of different interventions. In addition to increasing well-being, three of the PPIs were also effective at reducing negative outcomes, such as perceived stress, depression, and anxiety (Table 3). In terms of training design and content, all of the PPIs are long (ranging from four to 12 weeks) with weekly modules that focus on one topic per week. The Promising PPI topics and exercises can be viewed in Table 4. However, it should be noted that while the four exemplar PPIs share commonalities that can help inform future PPI design, there were also differences in theories, features, and duration.

**Table 4:** The most promising PPI training topics and exercises.

Drozd et al., 2014, p. 380	Feicht et al., 2013, p. 2	Ivtzan et al., 2016, p. 1400	Schotanus-Dijkstra et al., 2017 ^a^
1. “Introduction - Content: About BD, positive psychology, and happiness test” 2. “Gratitude - Content: How happy people habitually notice and appreciate the positive in life - Exercises: ‘Three good things’ - Homework: Practice ‘three good things.’” 3. “Engagement and Pleasant Activities - Content: What do happy people do to have a good day? - Exercises: Make a list of pleasant activities; Plan pleasant activities for the next day - Homework: Carry out a pleasant activity” 4. “Character Strengths - Content: About character strengths and their practical use - Exercises: Identify personal character strengths - Homework: Find new ways of using character strengths” 5. “Acts of Kindness - Content: Acts of kindness and how they influence well-being: - Exercises: Plan three kind acts - Homework: Carry out acts of kindness” 6. “Gratitude - Content: The pleasant life involves positive emotions about the past, present and future - Exercises: ‘Three good things’ - Homework: Write a gratitude letter” 7. “Mastery and Reattribution - Content: How to deal with adversity? - Exercises: Instructions for expressive writing - Homework: Write about a negative event the next four days Cognitive restructuring (‘ABCDE’ exercise)” 8. “Optimism - Content: Optimism in everyday life and its effects on mental and physical wellbeing - Exercises: ‘Best Possible Life’”	1. “Basic Principles (i). How do you feel? Check your state of mind.” (ii) What hindered you in the past from being happy? (iii) Write a happiness-diary! Note three things that made you happy today.” 2. “Joy of Community (i) Get some body’s contact in a way that is comfortable for you. (ii) Identify your best friends and meet them this week. (iii) Write a thank-you letter.” 3. “Joy of Luck (i) Tell three people your wishes. (ii) Rejoice somebody by doing an unexpected favor. (iii) Let fortuity decide to do something new and give favorable opportunities a chance.” 4. “Joy of Pleasure (i) Eat a meal mindfully. (ii) Be mindful and capture happy moments with your camera. (iii) Challenge yourself with exercises/sports.” 5. “Joy of Flow (i) Identify your strengths. (ii) Use them in a new way.” 6. “Joy of Bliss/Beauty (i) Give little presents to make somebody happy. (ii) Write a gratitude-diary and note three things a day you are thankful for. (iii) Enjoy ten minutes of silence every day.”	1. “Self Awareness - Video: Introduction to mindfulness, self awareness, positive psychology and meditation - Meditation: Introductory meditation focusing on awareness of breath, body and emotions - Daily practice: Keeping aware of thoughts and reactions throughout the day” 2. “Positive Emotions - Video: Discussion of the benefits of positive emotions and gratitude - Meditation: Gratitude meditation focusing on who or what one appreciates - Daily practice: Expressing gratitude for positive situations’ 3. “Self-compassion - Video: Explanation of the self-compassion concept, research review and methods to increase self compassion - Meditation: Adapted version of Loving Kindness meditation focusing on self compassion (Neff and Germer, 2013) -Daily practice: Replacing internal criticism with statements of kindness” 4. “Self-efficacy - Video: Introduction to character strengths and self-efficacy including enhancement methods - Meditation: Meditation focusing on a time when participant was at his/her best and using character strengths - Daily practice: Completing the Values in Action (*VIA*) character strengths survey and using strengths” 5. “Autonomy - Video: Introduction to autonomy and its connection with well-being - Meditation: Meditation on authentic self and action - Daily practice: Taking action in line with one’s values and noticing external pressure on choices”	1. “Positive Emotions - Diary of pleasant emotions: What happened, who was there, what did you feel, what did you think? - Three good things: Think about three things that went well today and savor those moments.” 2. “Discovering Strengths - Overview of your strengths: Which of the 47 strengths do you have and which of these give you energy and pleasure? - Identify your strengths I: Answer the 10 questions (ie, who inspires you?) that will help you to discover your strengths. - Identify your strengths II: Which strengths do you recognize in answering the 10 questions? - Vision of others: Ask 3–5 people about your top 5 strengths with examples from daily life. - Top 5 strengths: Based on all previous exercises, choose your top 5 strengths that also give you energy and pleasure.” 3. “Use of Strengths, Flow - Change ‘must’ into ‘want’: Make a list of things you do not like but must do. What are underlying intrinsic motivations? - Flow: Have you experienced flow and why? - Flow at the moment: How much flow did you experience the preceding week? When, how? - Challenge yourself: How can you create more flow in your life? Use your strengths in a new way.” 4. “Optimism, Hope - ABC-Diary: What do you think and do when something negative happens? How can you challenge favorite pessimistic thoughts? - Imagine your best possible self: Visualize yourself in the personal, relational, and professional domain.” 5. “Self-compassion - Wish yourself something good: Be mindful and identify your greatest need at this moment. Use your inner voice to repeat your compassionate wish. - Develop a compassionate inner voice: Write 5 min about situations in the preceding week wherein you showed self-compassion.”	9. “Flow - Content: How to use flow to create engagement and intrinsic motivation - Exercises: Identify personal flow activities; Plan a flow activity Homework: Carry out a flow activity” 10. “Gratitude - Content: How to enjoy small everyday moments of pleasure - Exercises: Instructions on how to share and savor small positive’ moments and to be proud of your achievements” 11. “Adaptation and Attribution - Content: How people (e.g., lottery winners) quickly adapt to their situation - Exercises: Instructions for attributing success to stable, global, personal characteristics, and failures to temporary, specific, situational characteristics” 12. “Stress and Mindfulness - Content: How prolonged stress can affect mental and physical well-being - Exercises: Practice mindfulness by focusing on one’s breathing” 13. “Summary - Content: Happiness test and blueprints for increasing well-being - Exercises: Summary of important tasks and exercises”	7. “Final (i) Detect your favorite happiness exercises. (ii) Be a happiness messenger and tell your favorite exercises to other people. (iii) Reward yourself for your happiness-work during the last week and give yourself a treat.”	6. “Meaning - Video: Discussion of meaning and wellbeing. Completion of writing exercise, Best Possible Legacy adapted from obituary exercise (Seligman et al., 2006) - Meditation: Meditation on future vision of self, living one’s best possible legacy Daily practice: Acting according to best possible legacy. Choosing meaningful activities” 7. “Positive Relations with Others - Video: Discussion of benefits of positive relationships and methods for relationship enhancement - Meditation: Loving Kindness Meditation - Daily practice: Bringing feelings of loving kindness into interactions” 8. “Engagement/Conclusion - Video: Introduction to engagement and savouring and their connection with positive emotions - Meditation: Savouring meditation focusing on food - Daily practice: Using savouring to engage with experiences - Conclusion: Summary of the program. Discussion of personal growth and invitation to keep meditating”	6. “Resilience - Coping style: Take the test to identify your prominent coping style(s). - Expressive writing: Write 15 min on at least 4 days about emotions, thoughts, and feelings around a negative or positive event. - Needs: What are your specific needs at this moment? Who should know your needs?” 7. “Positive Relations (I) - Active-constructive responding: Respond positively to good news shared by others. Use active communication skills, how does the other react? - Listen compassionately: Try to use elements of compassionate listening, such as ‘What feelings and needs does the other express?” - Expressing gratitude: Write a gratitude letter and/or read it aloud to the person you are thankful to.” 8. “Positive Relations (II) - Relaxation/meditation: Relax by doing a ‘body scan’, physical exercise, or ‘stand like a tree’. - Reflect on your needs: What are your intrinsic goals, needs and motives? Do you live those needs and why (not)? - Acts of kindness: Rejoice somebody by performing an unexpected act of kindness or by doing volunteer work.”

a *Description from* Schotanus-Dijkstra et al. (2015)*, pp. 6–7.*

Although all four exemplar studies were among the highest-quality RCTs in our sample, there were some methodological limitations present. All four studies used samples that were subject to self-selection bias and consisted of mostly educated females. All four RCTs also used waitlist control groups, which can create expectation effects, and all experienced participation attrition. In addition, all four studies used self-report measures although one study (Feicht et al., 2013) also used objective measures. Finally, long-term follow-up measurement was lacking with the longest follow-up measurement at 12 months (see Table 3).

Experimental evidence of the highest quality suggests these PPIs may be promising exemplars for future intervention design during the global pandemic and beyond, and seem promising for future implementation and evaluation in non-WEIRD contexts. However, it is important to emphasize that future PPIs guided by the findings of these exemplars should also be tested in a rigorous manner to make sure they are also efficacious and effective for more diverse populations in need, including populations in non-WEIRD countries. We acknowledge although we have identified some of the most valid causal evidence available for generating well-being with PPIs, the samples used in the most rigorous studies were not as diverse as we would have liked to be confident these PPIs will naturally generalize to different populations and non-WEIRD contexts. Nevertheless, we have identified the most promising causal evidence for guiding the design of PPIs for non-WEIRD countries, with appropriated adaptations to fit the specific context.

## Discussion

The aim of this review was to review existing systematic reviews and meta-analyses and identify the most promising PPIs for generating well-being based on the most rigorous experimental evidence available in the peer-reviewed literature. Four exemplar PPIs were identified from these meta-analyses, all of which were MPPIs in the form of self-administered training that can be administered to teach a variety of positive psychology topics and skills over the course of multiple weeks that participants can use to improve their well-being.

### Implications and Recommendations

The findings of meta-analyses as well as the most promising PPIs identified by this review provide a base of scientific evidence to inform the future design of PPIs for generating well-being in both pandemic and non-pandemic times. A major advantage of examining the distributions of PPI effects across many rigorous RCTs is that it provides a good sense of what one might expect when designing or replicating a PPI to generate well-being. It also provides some conditions of the format and study design that may bolster or diminish effects. Although using a no-intervention or placebo control group and having a lower-quality study may lead to greater effects, these are not the type of takeaways we hope designers replicate. Instead, we hope these findings underscore the importance of designing a high-quality PPI so as to achieve effects even with a strong active comparison group and high-quality study design.

Looking at specifics of design by “following the science,” MPPIs can be administered as a training to help people improve their own well-being by giving them knowledge and skills that will support them in daily life. The most promising PPIs we found illustrated that providing opportunities to learn, practice, reflect, relate, and plan can help ensure effectiveness (see Table 5 for a detailed description).

**Table 5:** Five components that can be incorporated into PPI design.

PPI Component	Objective	Description
Learn	Knowledge and awareness	Develop an awareness and understanding of topics and oneself.
Practice	Behavioral skills	Practice simple skills and exercises that can be incorporated into daily life.
Reflect	Sense-making and reinforcement	Practice reflection after exercises to encourage sense-making and reinforcement of new skills.
Relate	Engagement and accountability	Clarify understanding with experts and relate to peers to amplify effects and reinforce accountability.
Plan	Sustainability	Set goals and create a plan to practice new skills in daily life to encourage long-term sustainability.

When designing a training, a variety of topics, exercises, and skills based on the science of positive psychology and MPPIs can be provided to target multiple dimensions of well-being, both hedonic and eudaimonic. Since MPPIs can also decrease stress, depression, and anxiety (Hendriks et al., 2020), the reduction of these symptoms can also be targeted to help people who may be struggling with these symptoms during the pandemic. A design that incorporates mutually reinforcing activities can also amplify positive effects (Rusk et al., 2018). For example, incorporating the practice of mindfulness can enhance and sustain the positive benefits of positive psychology training (Ivtzan et al., 2016).

Successful interventions appear to be informed by scientific evidence and are tailored to fit the specific needs and contexts of participants (Donaldson and Chen, 2021). The most promising PPIs identified by this review can provide ideas for designing a curriculum (see Tables 3–5) and PPI meta-analyses point to theories and activities that have been shown to improve well-being across many studies (see Table 1), such as practicing gratitude (Davis et al., 2016; Dickens, 2017), kindness (Curry et al., 2018), mindfulness (Lomas et al., 2019; Slemp et al., 2019), and best possible self (Carrillo et al., 2019), as well as job crafting, strengths, and PsyCap in the workplace (Donaldson et al., 2019a). The curriculum of these interventions can be adapted to fit the needs and contexts of participants, including those from non-WEIRD countries.

Other aspects of intervention design can also be tailored to suit participants’ needs and contexts. Flexibility can encourage adherence and help meet a variety of participant needs and motivations across different contexts. Participants can choose where and when they complete the modules based on their schedule or tailor their learning by choosing the topics or activities that resonate with them. Longer PPIs have been found to be more effective than shorter ones (Koydemir et al., 2020), yet a large amount of time does not need to be devoted to activities to be effective as demonstrated by the four most promising PPIs. Providing flexibility can be helpful for people with heavy workloads, like frontline and essential workers, or parents who are working from home while balancing childcare responsibilities. Similarly, giving individuals the opportunity to self-select by engaging in activities that are more intrinsically motivating or well-suited can amplify the positive effects (Deci and Ryan, 2000; Sin and Lyubomirsky, 2009; Lyubomirsky and Layous, 2013). Providing reminders and opportunities to check progress can also be added to further encourage adherence and engagement.

Within the context of a global pandemic, the delivery mode of PPIs is an important consideration. For example, face-to-face interactions may no longer be as feasible to implement in a pandemic-impacted world. Online PPIs, particularly automated online self-help interventions, can be used while social distancing and implemented cost-effectively on a larger scale than face-to-face interventions (Muñoz, 2010). Although individualized interventions tended to show greater effects than self-help or group interventions across meta-analyses (Sin and Lyubomirsky, 2009; Weiss et al., 2016; Carr et al., 2020), three out of four of the most promising PPIs were online and were all self-administered with success. Some research has found many searching for well-being programs tend to be inclined to seek online PPIs (Parks et al., 2012, p. 1). A combination of automated content supplemented by live expert or peer support can also be considered. Although online interventions can reach more people, it is important to recognize that a “global digital divide” exists where access to technology is a barrier for those from lower socioeconomic backgrounds (Pick and Azari, 2008, p. 1). Therefore, in non-WEIRD countries that may have larger populations from lower socio-economic backgrounds, alternative modes of delivery can be considered to make PPIs accessible to those who lack adequate access to technology and the Internet. Physical self-help lessons or workbooks can be used and supplemented by additional guidance and support *via* email (Schotanus-Dijkstra et al., 2017). These materials can be mailed to meet social distancing guidelines and if participants do not have access to email, support can be provided *via* telephone.

Finally, we recognize that a multi-week training will not be feasible for everyone, especially those heavily impacted by the pandemic. The science of positive psychology also points to several effective smaller-dose mono-PPIs that can be used by anyone at any time. For those lacking time and resources, our recommendations based on the most promising PPIs and PPI meta-analyses provide simple yet effective exercises that anyone can try.

### Strengths and Limitations

This review makes several contributions to the positive psychology literature. First, we focused on the most rigorous research of PPIs, in the form of high-quality RCTs, using some of the most valid causal evidence available to identify the most promising PPIs for generating well-being. Second, this is the first systematic review of PPIs that makes use of the exemplar method. The exemplar approach is naturally aligned with the spirit of positive psychology, identifying exemplars in the upper bounds of what is possible as opposed to being limited by what is typical. We hope this unique approach can also serve as a model for future reviews in this field. Third, we believe this review will serve as an especially useful resource for practitioners since it provides practical, evidence-based recommendations for designing effective PPIs that will generate well-being.

There are also several limitations that should be acknowledged. First, there are no clearly defined universal criteria for what constitutes an exemplar (Bronk, 2012). We defined our own criteria to identify exemplary PPIs, but there may be other approaches that could be further explored with the longer-term goal of achieving consensus on what constitutes exemplarity among PPIs that target well-being and the RCTs that test their efficacy. Furthermore, it should be noted that how exemplarity is defined in a study will also influence results (Bronk, 2012). Second, our inclusion criteria were limited to RCTs while inclusive of all PPI types and features, samples, well-being measures, and well-being theories. RCTs test efficacy under highly controlled conditions, but more research is needed to draw conclusions about effectiveness in real-world settings. The variability in our sample also means that the PPIs, RCTs, and effect sizes we looked at are not perfectly comparable. Therefore, further research is needed to confirm generalizability and replicability of our findings. Future reviews of PPI studies can also explore the use of narrower inclusion criteria and more homogenous samples to confirm efficacy. Future research is needed to further test the effectiveness of the most promising PPIs and our recommendations for designing PPIs in real-world settings, including different contexts and with different populations. Finally, the samples used to test the most promising PPIs were from WEIRD countries and were mostly White and female, demonstrating a need for rigorous scientific PPI studies to use more diverse samples that include more non-WEIRD countries. None of the four exemplars came from non-WEIRD countries since there were fewer non-WEIRD studies to include and more non-WEIRD studies were rated lower-quality, even though they showed greater effect sizes in meta-analyses (Hendriks et al., 2018, 2020; Carr et al., 2020).

### The Importance of DEI for the Future of PPI Science

Our findings are consistent with previous research that found that the majority of RCTs on PPIs were conducted in WEIRD countries on samples that were mostly highly educated with a higher income (Hendriks et al., 2019). Among the RCTs identified in this review, there was also no mention of diversity, equity, or inclusion in the titles or abstracts of these papers. Positive psychology has been criticized for not attending much to issues of diversity, equity, and inclusion. Rao and Donaldson (2015) found that although women are overrepresented as participants in empirical studies, they are underrepresented as first authors, and discussions of issues relevant to women and gender are relatively scarce. Further, empirical research studies conducted across the world are based largely on White samples, and there is little research focused on race and ethnicity or individuals at the intersections of gender, race, and ethnicity. Rao and Donaldson (2015) suggested pathways for addressing these deficits and encouraged future positive psychology researchers to seek a better understanding of DEI issues related to positive psychology. Harrell (2018) and Pedrotti and Edwards (2017) extended this seminal DEI work and provided additional frameworks for understanding positive psychology concepts and interventions in cultural context, with diverse and marginalized groups, and with a focus on collective well-being. Warren et al. (2019) provided a detailed conceptual map for navigating and planning future research on well-being and flourishing through positive diversity and inclusion behaviors and practices. These prior efforts to encourage more emphasis on DEI are useful guides for adapting the most efficacious PPIs we found in this paper to meet the specific needs of the marginalized and vulnerable populations. But we would also like to point out that we cannot assume that the promising PPIs we have identified will necessarily have the same effects. Future efforts to examine PPIs in diverse, marginalized, vulnerable populations, and in non-WEIRD contexts are sorely needed to better understand how to reduce disparities and generate well-being for all (Bolier et al., 2013; Curry et al., 2018; Hendriks et al., 2018).

## Conclusion

We followed the positive psychology intervention science and discovered that the most rigorously tested PPIs clearly suggest how we might generate well-being in global pandemic and non-pandemic times. These experimental findings provide us with causal evidence that medium and longer-term well-being outcomes can be achieved with PPIs. It has also revealed the conditions under which PPIs are most likely to be effective and underscored the importance of conducting more rigorous PPI research in non-WEIRD contexts and designing the next generation of PPIs to better serve diverse, marginalized, and the underserved populations who are most likely to be the most negatively affected by a global pandemic.

## Data Availability Statement

The original contributions presented in the study are included in the article/supplementary material, and further inquiries can be directed to the corresponding author.

## Author Contributions

All authors listed have made a substantial, direct, and intellectual contribution to the work, and approved it for publication.

## Conflict of Interest

The authors declare that the research was conducted in the absence of any commercial or financial relationships that could be construed as a potential conflict of interest.

## Publisher’s Note

All claims expressed in this article are solely those of the authors and do not necessarily represent those of their affiliated organizations, or those of the publisher, the editors and the reviewers. Any product that may be evaluated in this article, or claim that may be made by its manufacturer, is not guaranteed or endorsed by the publisher.
